# Mesenchymal Stromal Cells Preconditioning: A New Strategy to Improve Neuroprotective Properties

**DOI:** 10.3390/ijms23042088

**Published:** 2022-02-14

**Authors:** Giovanni Schepici, Agnese Gugliandolo, Emanuela Mazzon

**Affiliations:** IRCCS Centro Neurolesi “Bonino-Pulejo”, Via Provinciale Palermo, Contrada Casazza, 98124 Messina, Italy; giovanni.schepici@irccsme.it (G.S.); agnese.gugliandolo@irccsme.it (A.G.)

**Keywords:** mesenchymal stromal cells, preconditioning, neurological diseases, neurogenesis, transplant

## Abstract

Neurological diseases represent one of the main causes of disability in human life. Consequently, investigating new strategies capable of improving the quality of life in neurological patients is necessary. For decades, researchers have been working to improve the efficacy and safety of mesenchymal stromal cells (MSCs) therapy based on MSCs’ regenerative and immunomodulatory properties and multilinear differentiation potential. Therefore, strategies such as MSCs preconditioning are useful to improve their application to restore damaged neuronal circuits following neurological insults. This review is focused on preconditioning MSCs therapy as a potential application to major neurological diseases. The aim of our work is to summarize both the in vitro and in vivo studies that demonstrate the efficacy of MSC preconditioning on neuronal regeneration and cell survival as a possible application to neurological damage.

## 1. Introduction

Mesenchymal stromal cells (MSCs) are known for their immunomodulatory and multipotent differentiation properties. The identification of MSCs requires different biomarkers [1]. According to the International Society for Cellular Therapy (ISCT), the minimum criteria to identify MSCs include: their plastic adherence under standard culture conditions; their ability to differentiate in vitro towards adipocytes, osteoblasts and chondroblasts; the expression of the surface molecules CD90, CD105 and CD73 and the lack of the hematopoietic markers CD34, CD45 and CD14, CD79α or CD19 and HLA-DR [2,3]. MSCs were initially isolated and identified from bone marrow (BM), which represents a rich source of MSCs. However, the painful process of collecting BM-derived MSCs (BM-MSCs) makes their use limited. Therefore, MSCs derived from other tissues may have other advantages compared to BM-MSCs [4,5]. Indeed, alternative sources of human MSCs (hMSCs), including umbilical cord (UC), adipose tissue (AT), birth-derived tissues, dental tissues and others, have been identified [6]. AT can be an important candidate for tissue engineering and MSCs-based therapies thanks to both the high hMSCs yield and easier collection procedures [6]. Moreover, given the young origin, perinatal tissues, such as the amnion, chorion and UC tissue Wharton’s jelly (WJ), could often be used as important sources of MSCs [7]. Dental-derived MSCs originate from the neural crest and are used as a source of MSCs for neurological applications [8,9].

MSCs have been used as important players in regenerative therapies for their proliferative capacity, immunomodulatory and antiapoptotic properties, multilinear differentiation ability and paracrine effects [10,11].

MSCs can modulate several effector functions through the interaction with innate and adaptive immune cells. Several studies reported that MSCs can migrate to damaged tissues to inhibit the release of inflammatory cytokines and promote the survival of damaged cells [12]. Moreover, MSCs can promote tissue regeneration following injury by reducing inflammation, increasing trophic factors, including brain-derived neurotrophic factor (BDNF), glial cell-derived neurotrophic factor (GDNF), vascular endothelial growth factors (VEGF) and nerve growth factor (NGF), as well as by recruiting local progenitor cells to replace damaged cells [12,13].

MSCs originate from the mesoderm but possess multilineage differentiation capacity [14], including towards endodermal and ectodermal cells [1]. It has been shown that, under favorable conditions, MSCs can differentiate into pancreatic β cells, cardiomyocytes, hepatocytes and neurons [7].

Moreover, it is now well-known that beneficial MSCs’ properties depend at least in part on their paracrine effects. In this regard, MSCs can release trophic and growth factors, microRNAs, cytokines and extracellular vesicles (EVs), which, together, constitute the secretome [11,15,16].

Consequently, the MSCs’ regenerative properties make MSCs therapy potentially useful to repair damaged tissues [17,18]. Furthermore, the secretome was shown to be efficacious in preclinical studies of neurological and neurodegenerative diseases, and it could be helpful for the development of new therapeutic approaches [16,19].

Although promising, MSCs can lose biological functions after isolation and in vitro expansion for a long time. MSCs can find a hostile environment and die after injection into damaged tissues. Consequently, to improve the efficacy of MSCs transplantation in regenerative medicine, it is necessary to find new strategies that allow the preservation of MSCs’ characteristics, and preconditioning with physical, chemical and biological factors may be an option [20].

The aim of this review is to summarize the evidence from experimental studies that highlight the efficacy of preconditioning in improving MSCs’ features and the application of preconditioned MSCs and derived secretome as potential therapeutic applications for neurological diseases.

## 2. MSCs Preconditioning in Neurological Diseases

Neurological diseases (ND) affect the nervous system and represent a large public health challenge, with a tremendous burden for individuals and their families, reducing the quality of life. ND may include different disorders with a sudden onset or progressive conditions, such as stroke, traumatic brain injury (TBI), Parkinson’s disease (PD), Alzheimer’s disease (AD), multiple sclerosis (MS), amyotrophic lateral sclerosis (ALS) and Huntington’s disease (HD). They are characterized by a progressive neuronal loss that can lead to cognitive and memory deficits. The progressive neuronal deterioration determines the axonal and synaptic degeneration, thus influencing the neurogenesis and plasticity of the neural stem cells (NSCs). MSCs and their secretome may be a promising treatment for ND [21].

Several studies suggested that the development of ND is caused by multifactorial conditions, including abnormal protein aggregation, neuroinflammation, oxidative stress, aging, mitochondrial dysfunction, autophagy as well as exposure to toxic compounds, such as metals and pesticides [22].

Therefore, the lack of effective treatments for ND led researchers to look for additional therapeutic tools, such as stem/stromal cells (SCs) [23]. Although different types of SCs have been used, MSCs therapy poses an interesting challenge in terms of safety, variability and ethical concerns compared to other SCs [14,24,25].

MSCs can differentiate into neural precursors or mature neurons, expressing neural markers that include nestin, microtubule-associated proteins-2, beta-III tubulin and neuron-specific enolase [26,27]. Different protocols were developed to induce neuronal differentiation of MSCs [28,29,30].

MSCs can be considered as interesting candidates to develop new therapeutic approaches to counteract ND thanks to these properties, together with the immunomodulatory capacity and the possibility to be isolated by autologous and allogeneic sources [26,31,32].

Neuroprotective effects of MSCs transplantation have been demonstrated in several experimental studies of neurodegenerative diseases. Indeed, it has been reported that AT-derived MSCs (AT-MSCs) administration into superoxide-dismutase-1 (SOD1)-mutant transgenic mice, the familial ALS mouse model, delayed the loss of motor neurons as well as increased the number of motor neurons in the lumbar region and also the expression levels of GDNF and basic fibroblast growth factor (bFGF) [33]. The effects of MSCs transplantation were also shown in a mouse model of stroke, where UC-MSCs infusion improved neurological deficits and angiogenesis through Notch-1 signaling. Moreover, the in vitro results demonstrated that UC-MSCs co-culture with oxygen, and glucose deprivation (OGD) neurons favored VEGF-A secretion, associated with Notch-1 signaling [34]. It was shown that the engraftment of BM-MSCs overexpressing BDNF and VEGF in cerebral ischemia rats improved neurofunctional deficits [35].

In order to improve the regenerative, immunomodulatory and anti-inflammatory properties as well as their therapeutic potential for ND treatment, different studies evaluated the effects of MSCs preconditioning. Indeed, it has been shown that the preconditioning of MSCs could be useful to improve cell survival and the resistance to insults induced by pathologies [36]. In this regard, it has been reported that proinflammatory stimuli, including cytokines, hypoxia, toxins, reactive oxygen species (ROS) and pharmacological agents, may enhance cell differentiation as well as increase the migration and homing capacity of the transplanted cells in the lesion site [37]. Additionally, transplantation of preconditioned MSCs improved their paracrine effect, regenerative and reparative potentials. In detail, preconditioning with proinflammatory cytokines, including interferon- γ (IFN-γ), tumor necrosis factor- α (TNF-α), interleukin-1 beta (IL-1β) or interleukin-1 alpha (IL -1α), enhanced the immunosuppressive properties [38].

Preconditioning with the optimization of the culture conditions may be a key strategy to improve the MSCs function both in vitro and in vivo. Therefore, the overall effect of these procedures can contribute to improve the efficacy of MSCs transplantation in both regenerative medicine and tissue engineering [20,39].

In particular, hypoxic preconditioning is widely studied. Hypoxia, through the expression of hypoxia inducible factor-1 (HIF-1), can regulate signal transduction and several cellular processes. HIF-1 is a heterodimeric α/β protein composed of constitutively expressed aryl hydrocarbon receptor nuclear translocator (ARNT) and the hypoxic response factor HIF-1α regulated by cellular oxygen concentration [40]. Hypoxia plays an important role in physiological processes. In particular, low levels of oxygen have proved to be involved in embryogenesis as signaling processes that regulate the proliferation and differentiation of SCs [41,42]. Indeed, changes in oxygen levels have been shown to be key factors for the generation of new neurons during neurogenesis [43].

## 3. The Effects of MSCs Preconditioning in ND Experimental Studies

Several preclinical studies performed both in vitro and in vivo reported the effects of preconditioned MSCs as a potential therapeutic application for the treatment of ND.

### 3.1. MSCs Preconditioning with Hypoxia

#### 3.1.1. MSCs Preconditioning with Hypoxia in In Vitro Studies

Hypoxic preconditioning was among the most investigated. In particular, different works evaluated its effects on MSCs differentiation and survival. Hypoxic preconditioning increased the size and number of neurospheres generated from BM-MSCs. This effect depended on epidermal growth factor (EGF), and, indeed, the inhibition of the EGF receptor attenuated the effects. However, hypoxia did not influence the capacity of neurospheres to generate neuron- or glia-like precursors. Interestingly, human Schwann cell-like cells obtained from hypoxia-treated BM-MSCs showed the ability to induce myelination in vitro [44].

Theus et al. evaluated the effects of hypoxic preconditioning in embryonic stem (ES) cell-derived neural progenitor cells (ES-NPCs) and BM-MSCs. The results of the in vitro study showed that sublethal hypoxia pretreatment protected against apoptosis of ES and BM-MSCs. Additionally, B cell lymphoma 2 (bcl-2), HIF-1α, erythropoietin (EPO) and EPO receptor were upregulated [45].

The beneficial effects of hypoxic preconditioning in neural induced-hMSCs (NI-hMSCs) were reported by Zhang et al. in vitro. Before inducing neural differentiation, hMSCs were incubated in hypoxic or normoxic conditions for 5 days. The results of the study reported that the hypoxic preconditioning promoted proliferation and the differentiation of NI-hMSCs into dopaminergic-like cells, as shown by the expression of the dopaminergic neuron marker nuclear receptor related 1 protein (nurr1) and the increase in tyrosine hydroxylase levels. Therefore, the study demonstrated encouraging results of preconditioned hMSCs, showing that they are capable to differentiate into dopaminergic-like neurons and may be helpful as possible therapy for tissue regeneration and locomotor recovery in PD [46].

Moreover, Pacary et al. observed in BM-MSCs that cobalt chloride (CoCl_2_) treatment, used to mimic low oxygen levels, promoted neuronal differentiation, probably through the HIF-1α involvement, as highlighted by the increase in EPO, VEGF and p21 expression levels. In detail, it was demonstrated that, 3 days following treatment with CoCl_2_, MSCs differentiated into neuron-like cells, as shown by the increased expression levels of nestin. Moreover, the mechanism by which CoCl_2_ promoted differentiation through HIF-1 was investigated. Indeed, the use of echinomycin, a molecule that inhibited HIF-1, blocked the neuroregenerative effect of CoCl_2_ on BM-MSCs. Conversely, treatment with Y-27632, a Rho kinase inhibitor, enhanced differentiation of BM-MSCs into neuron-like cells induced by CoCl_2_. Overall, the results demonstrated that BM-MSCs can differentiate into neuron-like cells in response to CoCl_2_, in part through the activation of HIF-1, and are potentiated by Rho kinase inhibition [47]. The same group showed that the inhibitor of Rho kinase also potentiated the action of deferoxamine, another HIF-1 inducer, in promoting the morphological changes of BM-MSCs toward neuron-like cells. Moreover, the authors confirmed that Y-27632, in addition to CoCl_2_, potentiates the neuronal differentiation of PC12 cells. Overall, the study proposed how a combined treatment targeting HIF-1 and Rho-kinase pathways could be useful for differentiating BM-MSCs into neuronal cells [48].

To show the effects of hypoxic preconditioning on MSCs neuronal differentiation, Gugliandolo et al. evaluated the transcriptional profile of gingival-derived MSCs (G-MSCs) induced toward neuronal differentiation in hypoxic and normoxic conditions using next generation sequencing. The transcriptomic analysis of hypoxic G-MSCs revealed a greater expression of genes involved in neuronal development and differentiation, and higher levels of nestin, PAX6 and GAP43 compared to the G-MSCs induced in normoxia. Therefore, the study showed that hypoxia improved the differentiation properties of MSCs [49].

Oxygen tension is an important environmental factor capable of influencing the neuroregulatory profile of MSCs. To confirm this, Teixeira et al. compared the analysis both of the secretome of human WJ-MSCs collected under hypoxic conditions and under normoxic conditions. The authors showed a different profile for the secretome of WJ-MSCs. In particular, it has been found that hypoxic preconditioning increased the secretion profile of the WJ-MSCs secretome compared to normoxic preconditioning. Specifically, 104 proteins were found in the cells cultured in normoxic conditions, whereas 166 proteins were found in WJ-MSCs in hypoxic conditions, but 81 proteins were in common. Both hypoxic and normoxic WJ-MSC secretomes were able to induce neuronal differentiation of neural progenitor cells (NPCs). WJ-MSCs’ secretome can improve differentiation and neuronal survival thanks to the presence of neuroregulatory molecules. In this regard, the secretome of WJ-MSCs collected from normoxic and hypoxic culture conditions can be important for the development of new therapeutic strategies, and the optimization of parameters, such as oxygen concentration, may improve the therapeutic effects of the secretome [50].

The studies evaluating the effects of hypoxia on neurodifferentiation and survival are resumed in Table 1.

Other studies evaluated the effects of hypoxic preconditioned MSCs and their secretome in co-culture with stressed cells.

The effects of hypoxia/reoxygenation (H/R) preconditioning in human BM-MSCs were investigated by Kim et al. The results of the study showed that, although BM-MSCs proliferation and viability decreased after one day of hypoxic culture, the subsequent reoxygenation of BM-MSCs for 5 days led to an increase in cell proliferation. H/R preconditioning enhanced cell migration and the expression levels of trophic factors, including VEGF, angiopoietin (ANG), fibroblast growth factor (FGF) and BDNF, as well as HIF-1α and phosphorylated AKT levels. To evaluate the beneficial effects of H/R preconditioning, the authors co-cultured hypoxic preconditioned BM-MSCs followed by long-term reoxygenation with cortical neurons of ischemic rat. The co-culture of H/R preconditioned BM-MSCs led to the survival of ischemic cortical neurons. Therefore, the study demonstrated a potential new strategy for the treatment of ischemic damage [51].

To show the efficacy of hypoxic preconditioning in BM-MSCs, Chen et al. performed an in vitro study in BM-MSCs subjected to simulated ischemia-reperfusion (I/R) injury. The results demonstrated that hypoxia increased the HIF-1α levels and was able to reduce apoptosis after I/R injury, inhibiting caspase-3 activation, especially in the experimental group subject with hypoxia of 8 h [52].

Pro-inflammatory microglial activation is a major cause of neuronal damage and progression of ND. High levels of inflammatory mediators at the expense of low levels of anti-inflammatory factors have been shown in cerebral I/R patients. Together with their paracrine factors, MSCs demonstrated therapeutic potential in ischemic and inflammatory diseases. Yu et al. evaluated in vitro the efficacy of hypoxic preconditioning BM-MSCs-conditioned medium (CM) on BV2 microglial cells exposed to OGD. The results of the study reported that hypoxic BM-MSCs-CM significantly reduced the damage in BV2 microglial cells and promoted cell survival. Furthermore, hypoxic BM-MSCs-CM reduced the inflammation in BV2 microglial cells evaluated through the reduction in inflammatory mediators, including TNF-α, IL-1β and interleukin-6 (IL-6), and upregulated the expression levels of anti-inflammatory cytokine interleukin-10 (IL-10), Arginase-1 and CD206. Therefore, the results showed that the hypoxic preconditioned BM-MSCs-CM is more effective in inducing the passage of microglia from an inflammatory phenotype to an anti-inflammatory one compared to BMMSCs-CM induced in normoxic conditions [53].

Luo et al. have also evaluated in vitro how the inflammatory environment can influence cell survival and the efficacy of BM-MSCs, culturing PC12 cells-CM and exosomes obtained from hydrogen peroxide (H_2_O_2_)-treated PC12 with BM-MSCs under oxidative stress. In particular, it was shown that the CM and exosomes of the PC12 cells favored the reduction in BM-MSCs cell viability. Hypoxia improved the cell survival of BM-MSCs through an increase in the HIF-1α expression [54].

The effects of hypoxic preconditioning on MSCs-EVs in post-stroke vascular remodeling were investigated by Gregorius et al. To evaluate the effects on cerebral angiogenesis in vitro, human cerebral microvascular endothelial cells (hCMEC/D3) exposed to OGD were cultured with EVs obtained from MSCs under hypoxic or normoxic conditions at various concentrations. The in vitro results showed that the EVs obtained from MSCs in hypoxic conditions promoted the increase in endothelial proliferation, cell migration and tube formation and survival following post-ischemic injury in a dose dependent manner. Moreover, the EVs obtained from MSCs under hypoxic conditions modulated the angiogenesis, as demonstrated by the miRNAs analysis in hCMEC/D3 cells, which revealed the modulation of miRNAs associated with neurotrophin signaling, focal adhesion, VEGF signaling, leukocyte transendothelial migration, adherens junction and cancer pathways. Among these miRNAs, the analysis demonstrated higher miR-126 levels, which is related to HIF-1α. The analysis of the preparations of EVs derived from hypoxic MSCs demonstrated an enrichment in growth factor pathway-associated proteins and extracellular matrix proteins/proteases and reduction in proteins involved in oxidative metabolism [55].

Lech et al. showed how optimizing the microenvironment can improve WJ-MSCs’ regenerative properties. The cells were encapsulated in a 3D hydrogel derived from human fibrin (FB) or platelet lysate (PL). It has been shown that these scaffold systems provide support, improving both the migratory capabilities and regenerative profile of WJ-MSCs. The WJ-MSCs were grown for a maximum of 7 days under two different conditions: 21% O_2_ and 5% O_2_. The study results reported an increase in the proliferation and survival of encapsulated WJ-MSCs grown with 5% O_2_. In addition, the increased expression of neural differentiation markers was shown. To assess the ex vivo neuroprotective properties of cell hydrogel constructs, an OGD model was used in hippocampal organotypic slice cultures (OHCs). When WJ-MSCs grown in scaffold were co-cultured with OGD-OHCs, a reduction in cell death in the CA1 region of the hippocampus was reported, and the WJ-MSCs cultured in the scaffold showed an increased mRNA expression of GDNF, transforming growth factor-β (TGF-β) 1 and VEGF-A. This effect was more prominent in co-culture with the WJ-MSCs grown in FB under 5% O_2_ conditions, where the up-regulated expression levels of BDNF and bFGF were found [56].

Huang et al. evaluated the effects of the hypoxic preconditioning of UC-MSCs in an in vitro cerebral I/R model. The hypoxic preconditioned UC-MSCs were co-cultured for 24 h with EVs derived from OGD/R-induced N2A neuronal cells. The results of the study demonstrated that hypoxic preconditioning improved the oxidative stress and cell survival of UC-MSCs. In conclusion, the hypoxic preconditioning could be a useful approach to improve the UC-MSCs therapy [57].

To show the effects of MSCs on the ischemic microenvironment, Zhuo et al. induced the OGD/R model in SH-SY5Y cells and exposed them to ischemic/hypoxic preconditioned olfactory mucosal-derived MSCs (OM-MSCs). A significant reduction in apoptosis and pyroptosis in insulted SH-SY5Y neurons, compared to the group co-cultured with OM-MSCs without pretreatment, was observed. The preconditioned cells showed a reduction in miR-181a, with the consequent upregulation of its targets GRP78 and Bcl-2 [58].

Hypoxic preconditioning modulated the cellular senescence of OM-MSCs exposed to hemin. Specifically, it was found that hypoxic preconditioning alleviated OM-MSC senescence through the upregulation of miR-326. Then, it was also demonstrated that miR-326 reduced the cellular senescence regulating autophagy through the polypyrimidine tract-binding protein 1 (PTBP1)/PI3K signaling [59].

Giacoppo et al. investigated the efficacy of hypoxic preconditioning on the secretome of human periodontal ligament stem cells (hPDLSCs) in scratch injured NSC-34 neurons. The results demonstrated that H-hPDLSCs-CM reduced inflammation and apoptosis. The H-hPDLSCs-CM contained NT-3, IL-10 and TGF-β [60].

Kwon et al. evidenced that hypoxic preconditioned human placenta-derived MSCs (HP-MSCs) showed an increase in the regeneration markers, including VEGF. The protective effects of HP-MSCs preconditioned with hypoxia were evaluated in CoCl_2_-induced retinal precursor R28 cells. Hypoxic HP-MSCs were able to increase the cell viability of CoCl_2_-induced R28 cells. Moreover, the co-culture with hypoxic HP-MSCs decreased the induction of HIF-1α, while regeneration markers GAP43, Thy-1 and VEGF increased in CoCl_2_-induced R28 cells. In particular, VEGF was shown to be an important mediator of the hypoxic preconditioned HP-MSCs’ recovery pathway [61].

The results discussed so far are resumed in Table 2.

#### 3.1.2. MSCs Preconditioning with Hypoxia in In Vivo Studies

Wei et al. reported, in an in vivo model of cerebral ischemia, the effects of hypoxic preconditioned BM-MSCs transplantation. The results of the study demonstrated that the transplantation of hypoxic preconditioned BM-MSCs, improved angiogenesis, neurogenesis and also the recovery of motor functions in the animals. In particular, sublethal hypoxia increased the expression of HIF-1α and trophic and growth factors, including BDNF, GDNF, VEGF, EPO and CXC motif chemokine receptor type 4 (CXCR4), in BM-MSCs. Therefore, hypoxic preconditioning through HIF-1α and trophic/growth factors overexpression led to a reduction in the microglial activation as well as an increase in neurogenesis and regenerative potential of BM-MSCs. The data obtained suggested that MSCs hypoxic preconditioning may be a useful therapeutic strategy to counteract the ischemic stroke [62].

The effects of CM obtained from hypoxia/normoxia preconditioned BM-MSCs were tested by Chang et al. in an experimental model of TBI. The results of the study showed that hypoxic preconditioning improved neurogenesis and reduced the damage induced by lesions in TBI rat brain compared to the experimental group treated with BM-MSCs cultured under normoxic conditions. Additionally, the rats treated with hypoxic preconditioned BM-MSCs secretome demonstrated better results in motor and cognitive tests. Overall, the results of the study still reported that hypoxic preconditioned MSCs showed an enhanced release of growth factors, such as VEGF, hepatocyte growth factor (HGF) and the HGF receptor c-Met, thus stimulating neurogenesis and improving the outcomes of TBI-induced injury [63].

The efficacy of the hypoxic preconditioning of BM-MSCs was reported by Wei et al. in a rat model of neonatal stroke. The results of the study demonstrated that BM-MSCs transplantation led to a significant reduction in infarct volume, promoting neurogenesis, angiogenesis and neurovascular repair, leading to an improvement in cerebral blood flow. Moreover, rats administered with BM-MSCs showed a greater recovery of sensorimotor functions and enhancement in the olfactory functions [64].

To demonstrate that hypoxic MSCs preconditioning significantly increases the neuroprotective effect of their CM, Roth et al. evaluated in vivo the effect of hypoxic BM-MSCs-CM in retinal ischemic injury rats. Hypoxic-CM showed a significant increase in 21 cytokines, including RAGE, ICAM-1, prolactin-R, IL-1 R6 and CINC-2α. CM from hypoxic-preconditioned BM-MSCs demonstrated a significant increase in nine proteins, such as VEGF, TIMP-1, MCP-1, ICAM-1 and CINC-1, compared to the non-hypoxic medium. Some of these proteins are negative regulators of apoptosis or cell death, while others regulate transcription, immune function, JAK-STAT and MAPK signaling, suggesting that the hypoxic BM-MSCs-CM can provide extensive protection from the degenerative mechanisms caused by ischemic damage. In the group treated with medium from hypoxic-preconditioned BM-MSCs, improvements in retinal function, decreased loss of the retinal ganglion cell layer and decreased apoptosis processes were noted compared to the control groups [65].

Chen et al. administered adult SD rats with transient focal cerebral ischemia with BM-MSCs cultured in normoxia or after hypoxic preconditioning. The preconditioned BM-MSCs led to a reduction in the volume of the infarct and improved neurological deficits compared to the control group. In particular, hypoxic preconditioned BM-MSCs reduced neuronal apoptosis in the ischemic brain and promoted cell differentiation and survival, as reported by the BDNF and VEGF levels [52].

The therapeutic potential of CoCl_2_ preconditioned BM-MSCs-CM was evaluated by Dai et al. in rats induced with perinatal hypoxia-ischemia (HI). CoCl_2_ preconditioned BM-MSCs showed increased HIF-1α levels, in association with an increase in EGF, EPO and IGF-1 in the CM. CoCl_2_ preconditioned BM-MSC-CM transplantation improved the spatial memory evaluated by Morris water maze following HI-induced damage. Moreover, it was also reported that CoCl_2_ preconditioned BM-MSC-CM improved brain damage and enhanced the long-term potentiation that is likely correlated with increased expression levels of GluR2 protein. In conclusion, the study reported that the paracrine effects of hypoxic preconditioned BM-MSCs could be useful to protect cells from HI brain damage [66].

The effects on the neuronal regeneration and functional recovery of the hypoxic preconditioning of MSCs were shown by Hu et al. in middle cerebral artery occlusion (MCAO) rats. The study results demonstrated that the transplantation of allogeneic rat BM-MSCs cultured in hypoxic conditions improved cell survival and neurological deficits. Moreover, it was reported that hypoxic preconditioning improved the cell survival of BM-MSCs in a CXC motif chemokine receptor type 12 (CXCR12)/CXCR4 signaling dependent manner. In this regard, it was shown that activation of the CXCR4 signal can mobilize MSCs in the ischemic area to promote neuronal regeneration [67].

Moreover, Luo et al. reported in SCI rats the effects of BM-MSCs preconditioned with hypoxia. The study results demonstrated that hypoxic preconditioning improved BM-MSCs survival, motor and behavioral deficits in SCI animals [54].

The effects of hypoxic preconditioned MSCs-EVs in MCAO mice were investigated by Gregorius et al. Hypoxic MSCs-EVs reduced neurodegeneration and improved neurological deficits and microvascular density in MCAO C57BL6/J mice [55].

Wang et al., to understand the effects of hypoxic preconditioning, performed a study on global cerebral ischemia rats administered intravenously with hypoxic preconditioned MSCs. The results of the study reported that MSCs transplantation promoted the increase in migration and cell survival as well as led to the reduction in neuronal apoptosis and inflammation in the cerebral cortex compared to the groups administered with MSCs cultured with normal O_2_ levels. Additionally, it was shown that hypoxic preconditioning promoted the migration and proliferation of MSCs through the involvement of signaling molecules, including HIF-1α, CXCR4 and PI3K/AKT [68].

To show the effects of MSCs transplantation on the ischemic microenvironment, Zhuo et al. induced the MCAO experimental model. Ischemic/hypoxic preconditioned OM-MSCs transplantation reduced the damaged infarct cortex and improved motor function in association with a reduction in apoptosis and pyroptotic cell death. Moreover, preconditioned OM-MSC transplantation enhances mitochondrial function recovery through miR-181a signaling, leading to the upregulation of target genes GRP78 and Bcl-2 [58].

The results of hypoxic preconditioning on MSCs transplantation were also evaluated by Liu et al. in intracerebral hemorrhage (ICH) experimental models. Intracerebral administration of hypoxia-preconditioned OM-MSCs in ICH C57BL/6 mice, 14 and 28 days post-implant, improved behavioral deficits and neuronal apoptosis compared to the control groups [59].

In order to confirm the neuroprotective effects of hypoxic preconditioning, Liu et al. treated ICH mice with normoxic/hypoxic preconditioned OM-MSCs. The intracerebral administration of hypoxia-preconditioned OM-MSCs led to the reduction in neuroinflammation, as reported by the attenuation of microglial pyroptosis, inflammatory cytokines and neuronal damage. Probably, hypoxic preconditioning led to the reduction in microglial pyroptosis through a mechanism mediated by NLRP3 inflammasome to promote the passage of microglia into the anti-inflammatory phenotype. Moreover, mice transplanted with preconditioned OM-MSCs showed an improvement in behavioral deficits [69].

The effects of MSCs preconditioning have also been demonstrated by Zhilai et al. in SCI rats. The UC-MSCs preconditioned with physioxya showed the up-regulation of trophic and growth factors, such as HGF, BDNF and VEGF. The transplantation of UC-MSCs preconditioned with physioxya improved the axonal regeneration, reducing inflammatory cell infiltration and apoptosis and improving functional recovery [70].

Giacoppo et al. investigated, in an experimental autoimmune encephalomyelitis (EAE) mouse model of MS, the efficacy of the secretome of hPDLSCs obtained in hypoxic preconditioning. The authors administered intravenously EAE mice with hypoxic preconditioned hPDLSCs-CM (H-hPDLSCs-CM) 14 days after the experimental model induction. The results of the H-hPDLSCs-CM administration demonstrated an improvement in the clinical symptoms in the MS experimental model, as shown by the test scores. It is noteworthy that the administration of H-hPDLSCs-CM led to a reduction in inflammation measured by the reduction in inflammatory cytokines and the marked expression of the anti-inflammatory cytokine IL-37 in mice. Furthermore, a reduction in oxidative stress, apoptosis and autophagy was observed after H-hPDLSCs-CM transplantation. Overall, the study reported that H-hPDLSCs-CM could be a useful strategy for MS [60].

Moreover, the effects of MSCs hypoxic preconditioning on neurological recovery were shown by Ma et al. in TBI rats. The results of the study demonstrated that the administration of AT-MSCs cultured under hypoxic conditions improved the spatial memory and motor deficits in TBI rats. The hypoxic environment modulated the growth, differentiation and survival of MSCs. Moreover, it was observed that the transplantation of AT-MSCs preconditioned with hypoxia led to an increase in the cell survival as well as a reduction in apoptosis and inflammation [71].

The involvement of VEGF in the regenerative properties of human placenta-derived MSCs (HP-MSCs) preconditioned with hypoxia was evaluated. Indeed, Kwon et al. reported that the hypoxic preconditioning of HP-MSCs, in an experimental model of optic nerve compression, repairs the axons of the optic nerve according to a mechanism that led to the upregulation of HIF-1α/VEGF [61].

The results discussed so far are resumed in Table 3.

### 3.2. The effects of MSCs Preconditioning with Chemical and Pharmacological Substances, Trophic Factors and Cytokines

#### 3.2.1. MSCs Preconditioning with Chemical and Pharmacological Substances, Trophic Factors and Cytokines in In Vitro Studies of Neurological Diseases

Several preclinical studies proposed the neuroregenerative and immunomodulatory effects of MSCs preconditioning with chemical or pharmacological substances, trophic factors and cytokines as a potential application for many ND.

It was shown in vitro that the valproic acid (VPA) preconditioning of hP-MSCs improved neural differentiation [72].

Since hMSCs neurogenic differentiation into NPCs could be a useful therapy for ND, nowadays, many techniques have been designed by researchers to improve the differentiation of MSCs into NPCs. In this regard, Sallam et al. developed a safe, effective and fast protocol to differentiate HS5 cells, a type of hMSC, with small molecules that act as chemical inducers. The effects of preconditioned MSCs with different inducers, including β-mercaptoethanol (BME), triiodothyronine (T3) and curcumin (CUR), were studied. To evaluate the effects of hMSCs preconditioning on the differentiation into NPCs, 24 h prior to neuronal induction, hMSCs complete medium was changed with pre-induction medium containing BME, T3 and CUR. Although the BME preconditioning promoted NPCs generation, its toxicity on cell viability compared with T3 and CUR limited its effects. Additionally, it has been shown that T3 and CUR could promote NSCs differentiation more efficiently than BME. Preconditioning promoted neurogenesis, proliferation and differentiation into NPCs of hMSCs, probably through the induction of key transcription factors involved in brain development and functioning, including PAX6, SOX2, DLX2 and GAP-43 [73].

Sun et al. evaluated the effects on ischemic injury and apoptosis of sevoflurane preconditioned MSCs in neuron-like PC12 cells cultured under hypoxia and serum deprivation (H/SD) conditions for 24 h. At first, the authors reported that MSCs preconditioning with sevoflurane for 2 h improved migration and cell survival but not cell proliferation in H/SD conditions. Furthermore, it was also shown that neuron-like PC12s were more resistant to H/SD damage when co-cultured with sevoflurane-preconditioned MSCs. Overall, the study results suggested that preconditioning with sevoflurane improved the therapeutic potential of MSCs in ischemic conditions, probably involving HIF-1α, HIF-2α, p-Akt/Akt and VEGF resulting in upregulation [74].

The neuroprotective effects induced by the preconditioning of WJ-MSCs were also shown by Kim et al. both in vitro and in vivo. The in vitro study demonstrated that thrombin preconditioning, which was reported to be better compared to the other preconditioning methods in promoting healing, significantly enhanced the antioxidant, antiapoptotic and anti-inflammatory effects of naive WJ-MSCs in OGD-induced primary cultures of rat cortical neurons. In particular, it was found that thrombin preconditioning improved the therapeutic efficacy of MSCs transplantation, mainly stimulating the secretion of growth and trophic factors, including VEGF and BDNF [75].

The results discussed so far are resumed in Table 4.

#### 3.2.2. MSCs Preconditioning with Chemical and Pharmacological Substances, Trophic Factors and Cytokines in In Vivo Studies of Neurological Diseases

The effects of multipotent MSCs transplantation were also shown by Babenko et al. in a stroke experimental model. To perform the study, multipotent MSCs were preconditioned via co-culture with rat cortical neurons, thus demonstrating the presence of intercellular communications that favor the interchange of cellular contents from one cell to another. In particular, neurons, through the formation of intercellular contacts, can transfer their cytosolic content to MSCs and may improve the paracrine effects of MSCs. Additionally, the mitochondrial transfer from MSCs to neurons was demonstrated. To show the effects of transplantation, MCAO rats were administered with an intravenous injection of MSCs co-cultivated with neurons and compared with control groups infused with a solution containing only neurons or MSCs, respectively. The results of the study demonstrated that the transplantation of MSCs co-cultivated with neurons led to a reduction in the volume of infarction in the brain and improved the brain functional recovery. MSCs co-cultured with neurons showed a slight increase in the BDNF levels, which was localized in vesicular structures adjacent to the plasma membrane. Therefore, the study demonstrated that MSCs preconditioned by coculturing with neurons may be useful to improve the recovery of damaged brain tissue post-stroke, also through intercellular crosstalk [76].

The effect of dimethyloxalylglycine was shown by Esmaeilzade et al. in an AD experimental model. Dimethyloxalylglycine preconditioning increased cell viability, migration and expression of CXCR4, CCR2, nuclear factor E2-related factor 2 (Nrf2) and HIF-1α. Additionally, in vivo dimethyloxalylglycine preconditioned BM-MSCs transplantation improved learning and memory deficits in the AD rats. An increase in BDNF and antioxidant capacity was found in the hippocampus of AD animals [77].

The effects of preconditioned MSCs-EVs, as well as the underlying neuroprotective mechanisms, have been also investigated by Chu et al. in neonatal mice induced with HI injury. The administration of hydrogen sulfide (H_2_S)-EVs (derived from NaHS-preconditioned MSCs) in neonatal HI model mice demonstrated an enhancement of neuroprotective and anti-inflammatory effects compared to animals treated with EVs derived from non-preconditioned MSCs. In detail, HI mice administered with MSCs-EVs preconditioned with H_2_S reported a reduction in the levels of pro-inflammatory mediators and less brain tissue loss. Similarly, the administration of EVs derived from H_2_S preconditioned MSCs improved cognitive and memory deficits in the animals. The beneficial effects of preconditioned EVs involved the upregulation of miR-7b-5p, which targets FOS implicated in cellular process, including cell proliferation, differentiation and survival. Indeed, H_2_S preconditioning was able to upregulate miR-7b-5p in EVs. Moreover, the miR-7b-5p levels increased in the ipsilateral cortex after H_2_S-EVs treatment. It was demonstrated that miR-7b-5p/FOS mediated the anti-inflammatory effects of H_2_S preconditioned EVs in acute HI injury [78].

Moreover, MSCs transplantation as a potential application to counteract inflammation that occurs in ND was shown by Losurdo et al. in AD 3xTg mice. To increase their anti-inflammatory capacity, strategies such as MSCs cytokines preconditioning could be useful to increase the release of immunomodulating EVs. In particular, MSCs were preconditioned with TNFα and IFNγ for 48 h and EVs collected. The study results demonstrated that the intranasal administration of MSCs-EVs reduced the inflammation, evaluated by the reduction in microglial activation, as well as improved the neuroprotective effects, as shown by the dendritic spines increase. Moreover, the authors showed the immunomodulatory effects of MSCs-EVs evaluated by using primary cultures of microglial cells. In this regard, it was found that the administration of MSCs-EVs suppresses the expression of inflammatory cytokines and favors the passage of microglia from an inflammatory to an anti-inflammatory phenotype [79].

The effects of MSCs preconditioning were also observed by Somoza et al. in a PD mouse model. When grown in a medium containing fetal bovine serum, human MSCs reduced BDNF induction. Conversely, being cultured in fetal bovine serum-free medium enriched with EGF and bFGF favored the increase in BDNF production. The transplantation of human MSCs into the injured brain of mice induced with 6-hydroxydopamine (6-OHDA) led to a significant increase in the activity of dopaminergic neurons, thus showing a neuroprotective effect of BDNF-secreting MSCs. A gene expression analysis revealed that preconditioned MSCs showed an increased expression of the genes involved in trophic effects, antioxidant, anti-apoptosis, cytokine/chemokine receptor, migration, cellular stress and mitochondrial energy pathways [80].

The efficacy of MSCs preconditioning has also been evaluated through the use of the pharmacological agents used as mood stabilizers, such as lithium and VPA, known for their neurotrophic and neuroprotective properties. Indeed, lithium and VPA have been shown to inhibit cellular glycogen synthase kinase-3 (GSK-3) and histone deacetylase (HDAC), thereby regulating the signaling pathways and molecules with neurotrophic and neuroprotective functions. GSK-3 is involved in several cellular processes, including signaling and cellular transport, glycogen metabolism, cell proliferation and migration, while HDAC inhibition could reduce apoptosis [81]. In this regard, Linares et reported in a mouse model of HD that lithium and VPA preconditioned BM-MSCs transplantation led to a reduction both in neuronal loss and formation of huntingtin aggregates. Moreover, the intranasal administration of preconditioned BM-MSCs with lithium and VPA combined also improved the neuronal survival and motor and behavioral deficits in the HD animal model compared to control groups [82].

Zhang et al. showed the effects of BM-MSCs preconditioned with sodium hydrosulfide, a H_2_S donor. The preconditioned cells showed an increase in proliferation and a reduction in apoptosis, as shown by a reduction in the Bax/Bcl-2 ratio in the hypoxia-ischemic condition. Preconditioning also increased BDNF and VEGF secretion. H_2_S exerted neuroprotective effects on cell proliferation and survival, probably through ERK1/2 and AKT signaling. It is noteworthy that treatment with NaHS reduced the level of HIF-1α, which was increased in hypoxia-ischemic-induced BM-MSCs. The results of the in vivo study showed that, 14 days after injection, preconditioned BM-MSCs reduced infarct volume and apoptosis, as demonstrated by the Bax/Bcl-2 ratio and increased BDNF and VEGF expression [83].

The therapeutic efficacy of preconditioned MSCs transplantation was also investigated by Min et al. in an experimental model of ICH rats induced with collagenase type IV. Twenty-four hours after the model induction, the rats were divided into experimental groups and treated with apocynin-preconditioned hPMSCs or naïve hPMSCs. The results of the study showed that the preconditioning of MSCs with apocynin, known to inhibit nicotinamide adenine dinucleotide phosphate (NADPH) oxidase, improved the therapeutic efficacy in the acute phase of ICH by exerting neuroprotective effects by strengthening the cerebral vascular system. In particular, it was shown that, compared to the naïve group, the preconditioning with apocyanin led to a reduction in the volume of cerebral edema and neurodegeneration 48 h after the insult. Moreover, apocynin-preconditioned hPMSCs promoted microvascular integrity, evaluated by a comparison of the tight junction expression levels [84].

Furthermore, in a PD mouse model induced with 6-OHDA, Kim et al. demonstrated that the transplantation of hPMSCs or hPMSCs-derived neural phenotype cells (hpNPCs), grown in proliferation medium containing FGF-4 and heparin, exerted beneficial effects, but hpNPCs demonstrated a greater protective effect with a longer lasting recovery in motor deficits. In particular, hpNPCs survived, differentiated into neurons at the grafted site and were also able to protect dopaminergic neurons against 6-OHDA induced cell death. The hpNPCs induced delta-like ligand (DLL)1 and neurotrophic factors able to modulate the environment to promote neuroprotection. Overall, both DLL1-contact signals and paracrine factors are useful for enhancing the neuroprotective mechanisms mediated by hpNPCs [85].

The efficacy of MSCs preconditioning was shown by Gao et al. in a mouse model of SCI. To improve the efficiency of cell therapy, the authors developed a transdifferentiation protocol. Human AT-MSCs were differentiated toward neuron/motoneuron-like cells by using growth factors, retinoic acid and sonic hedgehog for 24 h. The results of the study demonstrated that the transplant of motoneuron-like cells derived from AT-MSCs into the injury site improved cell survival, integration and differentiation into neuronal cells. Moreover, the preconditioning of the transplanted AT-MSCs led to a partial recovery of locomotor functions by restoring damaged neural circuits [86].

The effects of thrombin treated WJ-MSCs were evaluated in hypoxic ischemic encephalopathy (HIE) rats. The HIE rats intraventricularly transplanted with thrombin preconditioned WJ-MSCs showed improvements in inflammation and apoptosis, astroglial activation and sensorimotor functions [75].

The results discussed so far are resumed in Table 5.

## 4. Major Challenges Encountered in Clinic Setting of Mesenchymal Stromal Cells

Despite the success achieved in experimental studies, the use of MSCs shows some challenges that should be overcome before the clinical application of MSCs. In this regard, regenerative, anti-inflammatory and anti-apoptotic effects have been reported in preclinical studies, suggesting the use of MSCs as a potential application for the treatment of neurological and neurodegenerative diseases. However, their application in clinical practice requires new strategies to optimize their use [87]. Therefore, over the years, researchers tried to develop new strategies capable of overcoming the obstacles found in the clinical setting, including the number of cells available, the route of administration, immunorejection, cell engraftment and tumorigenicity [88]. Given the high proliferation rate, MSCs cannot be stably maintained for long periods. Moreover, it is not yet known whether single or multiple administrations are required for successful transplantation [88]. A further limitation regards the timing of administration and collection and the use of autologous or allogeneic sources [88,89].

Although several strategies tried to compare the efficacy and safety of the administration routes, the different pathologies and clinical indications do not currently allow having an optimal and standardized method for the MSCs administration. In this regard, it has been shown that the topical application or injection of MSCs to a specific site could be less invasive, useful for a precise administration and may improve cell engraftment [87,90]. Moreover, it has been reported that biomaterials may be useful to improve the regenerative properties and success of the MSCs transplant [91,92]. Moreover, intramuscular administration can be considered a safe and simple method compared to intraperitoneal and subcutaneous routes [93]. Of note, additional MSCs administration routes can be used for specific applications. Intranasal administration may be useful for the treatment of neurological and neurodegenerative diseases in order to avoid the risks associated with direct injection into the central nervous system (CNS) [94,95,96].

Moreover, MSCs-based transplantation therapy can lead to immunorejection problems in the case that the MSCs do not belong to the patient. Researchers are trying to devise strategies capable of evading immune recognition. Therefore, the use of biomaterials and techniques capable of protecting MSCs by immunogenicity and enhancing paracrine effects may be useful as applications to severe injury [91,97].

To date, there are no clinical studies that have reported tumorigenic events related to MSCs therapy. Consequently, MSCs treatment appears to be safe and tolerated in human patients [98,99]. To ensure that MSCs administered do not contain potentially tumorigenic transformed cells, the scientific community needs to investigate the potential malignant transformation of MSCs [99].

Although preclinical studies have generated great enthusiasm in tissue and regenerative medicine, before MSCs administration could be applied in clinical practice, it is necessary to clarify the mechanisms of action, standardization of culture methods and collection and administration of MSCs [100]. Methods such as preconditioning may improve the therapeutic properties of MSCs, increasing their survival and paracrine mechanisms, leading to an improvement also in their neuroprotective properties. Indeed, preclinical studies reported major beneficial effects of preconditioned MSCs. However, before applying MSCs preconditioning in the clinical setting, it is necessary, other than overcoming the basic problems of MSCs therapy, to standardize the culture conditions, which compound should be used, at which concentration to avoid toxicity and the time of preconditioning.

## 5. Conclusions

**Figure 1:**
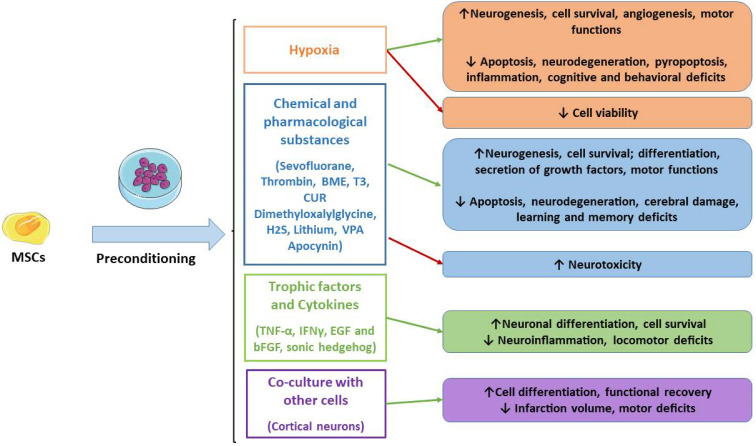
Main MSCs’ preconditioning methods. Green arrows indicate the advantages, while red arrows indicate disadvantages. MSCs: mesenchymal stromal cells; BME: β-mercaptoethanol; T3: triiodothyronine; CUR: curcumin.

For decades, research has focused on the improvements of MSCs’ application in regenerative medicine and for the treatment of ND. Therefore, strategies capable of enhancing MSCs’ survival, differentiation, regenerative, immunomodulatory and anti-inflammatory properties may be useful to improve MSCs transplantation to repair damaged tissues. In this context, the preconditioning through the optimization of the culture conditions with physical, chemical and biological agents may be helpful to enhance MSCs’ properties. In detail, this review offered an overview of the preclinical studies that demonstrated the therapeutic efficacy of preconditioned MSCs, including better neuroprotective effects in experimental models compared to naïve MSCs. In Figure 1, we reported the studied preconditioned methods.

It has been shown that the preconditioning of MSCs can promote migration, neuronal differentiation and the secretion of trophic factors, including BDNF, GDNF, VEGF, EPO and CXCR4. Depending on the preconditioning method, signaling pathways are modulated, such as HIF-1, leading to the inhibition of apoptosis and improving cell survival. In this way, preconditioned MSCs can reduce neuroinflammation and neuronal injury, leading to better cognitive and motor functions in experimental models.

However, even if MSCs preconditioning shows many positive aspects in experimental studies, it is necessary to develop standardized methods and culture conditions before clinical application.

## Figures and Tables

**Table 1:** Summary of in vitro studies that reported the effects of hypoxic preconditioning on neurodifferentiation and survival.

Co-Cultured Cells	MSCs	Preconditioning	Results	Mechanism	Ref.
NA	neurospheres generated from both rat and human BM-MSCs	Hypoxia 1% O_2_	↑ size and number of neurospheres	↑ EGFR	[44]
NA	BM-MSCs	Hypoxia 1% O_2_ and normoxia 21% O_2_	↓ apoptosis	↑ HIF-1α and EPO	[45]
NA	Neurally induced BM-MSCs	Hypoxia 4% O2 and normoxia 21% O_2_	↑ proliferation and neuronal differentiation into dopaminergic-like cells	-	[46]
NA	BM-MSCs differentiated into neuron-like cells	CoCl_2_ 100 μM	↑ neuronal differentiation	↑ HIF-1 ↓ Rho kinase	[47]
NA	BM-MSCs	Deferoxamine 150 μM	↑ neuronal differentiation	↑ HIF-1 ↓ Rho kinase	[48]
NA	G-MSCs differentiated in neuronal cells	Hypoxia 3% O_2_ and normoxia 21% O_2_	↑ neuronal development and differentiation	-	[49]
NPCs	WJ-MSCs secretome	Hypoxia 5% O_2_ and normoxia 21% O_2_	neuronal differentiation	Presence of neuroregulatory factors	[50]

BM-MSCs: bone marrow-derived MSCs; ↑: increase; ↓: decrease; EGFR: epidermal growth factor receptor; HIF-1: hypoxia inducible factor-1; EPO: erythropoietin; HIF-1α: hypoxia inducible factor-1α; CoCl_2_: cobalt chloride; G-MSCs: gingival-derived MSCs; NPCs: neural progenitor cells; WJ-MSCs: Wharton’s jelly-derived mesenchymal stromal cells.

**Table 2:** Summary of in vitro studies that reported the effects of neuroprotection on hypoxic preconditioned MSCs.

Cell and Stress	MSCs	Preconditioning	Results	Mechanism	Ref.
Cortical neurons of ischemic rats	BM-MSCs	H/R 1% O_2_	↑ migration, cell survival of cortical neurons	↑ HIF-1α, VEGF, ANG, FGF, BDNF and Akt	[51]
BV2 microglial cells cultivated in OGD conditions	BM-MSCs-CM	Hypoxia 1% O_2_	↑ cell survival ↓ neuronal damage and inflammation	↑anti-inflammatory microglia	[53]
PC12 cells H_2_O_2_ induced	BM-MSCs	Hypoxia	↑ cell survival	↑ HIF-1α	[54]
hCMEC/D3 exposed to OGD	MSCs-EVs	Hypoxia 1% O_2_ and normoxia 21% O_2_	↑ proliferation, cell migration, tube formation and survival	↑ miR-126	[55]
WJ-MSCs grown in scaffolds and co-cultured with OHC	WJ-MSCs	Normoxia 5% O_2_ and 21% O_2_	↑ proliferation and cell survival	↑ growth factors	[56]
SH-SY5Y neurons exposed to OGD/R	OM-MSCs	Hypoxia 3% O_2_	↓ apoptosis	↑ GRP78 and Bcl-2 through miR-181a	[58]
NSC-34 neurons exposed to scratch injury	hPDLSCs CM	Hypoxia 3% O_2_	↓ inflammation and apoptosis	↑ NT3, IL-10 and TGF-β	[60]
R28 retinal precursor cells exposed to CoCl_2_	HP-MSCs	Hypoxia 2.2% O_2_	↑ cell viability	↑ VEGF	[61]

BM-MSCs: bone marrow-derived MSCs; H/R: hypoxia/reoxygenation; ↑: increase; ↓: decrease; HIF-1α: hypoxia inducible factor-1α; VEGF: vascular endothelial growth factors; ANG: angiopoietin; FGF: fibroblast growth factor; BDNF: brain-derived neurotrophic factor; OGD: oxygen and glucose deprivation; BMMSCs-CM: bone marrow derived MSCs-conditioned medium; H_2_O_2_: hydrogen peroxide; hCMEC/D3: human brain microvascular endothelial cells; MSCs-EVs: MSCs-derived extracellular vesicles; WJ-MSCs: Wharton’s jelly-derived mesenchymal stromal cells; OHC: organotypic slice cultures; UC-MSCs: umbilical cord-derived mesenchymal stromal cells; EVs: extracellular vesicles; OM-MSCs: olfactory mucosal-derived MSCs; bcl-2: B cell lymphoma-2; hPDLSCs: human periodontal ligament stem cells-conditioned medium; IL-10: interleukin-10; TGF-β: transforming growth factor-β: CoCl_2_: cobalt chloride; HP-MSCs: human placenta-derived MSCs.

**Table 3:** Summary of the in vivo studies that reported the effects of hypoxic preconditioning of MSCs.

Model/Species	MSCs	Preconditioning	Results	Mechanism	Ref.
MCAO/rats	BM-MSCs 1 × 10^6^	Hypoxia 0.5% O_2_	↑ angiogenesis, neurogenesis and motor functions	↑ HIF-1α and trophic/growth factors	[62]
TBI/rats	BM-MSCs 100 μg of CM/kg	Hypoxia 0.5 % O_2_ and normoxia 21% O_2_	↑ neurogenesis ↓ brain damage, motor and cognitive deficits	↑ VEGF, HGF and HGF receptor c-Met	[63]
Neonatal stroke/rats	BM-MSCs 1 × 10^6^ cells	Hypoxia 0.1–0.3% O_2_	↑ neurogenesis, angiogenesis, sensorimotor and olfactory functions ↓ infarct volume	-	[64]
Retinal ischemia/rats	BMMSCs-CM at a final dose of 4 μL	Hypoxia 1% O_2_	↓ apoptosis and neurodegeneration	↑ VEGF, TIMP-1, MCP-1, ICAM-1 and CINC-1	[65]
Transient focal cerebral ischemia/rats	BM-MSCs 2 × 10^6^	Hypoxia 1% O_2_	↓ volume of the infarct and neurological deficits	-	[52]
HI/rats	BM-MSCs CM 1.0 μl/h for 7 days	Hypoxia 0.4% to 2.3% O_2_	↓ neurodegeneration and cognitive deficits	↑ HIF-1α, VEGF	[66]
MCAO/rats	BM-MSCs	Hypoxia 0.5% O_2_	↑ neuronal regeneration ↓ neurological deficits	↑ CXCR12/CXCR4 signaling	[67]
SCI/rats	BM-MSCs 2.0 × 10^6^ cells	Hypoxia	↑ cell survival, motor and behavioral deficits	-	[54]
MCAO/mice	intravenous administration of MSCs-EVs	Hypoxia 1% O_2_ and normoxia 21% O_2_	↓ neurodegeneration and neurological deficits	-	[55]
Global cerebral ischemia/rats	BM-MSCs 4 × 10^7^ in 0.5 mL of PBS	Hypoxia 1% O_2_	↑ migration and survival cell ↓ apoptosis and neuroinflammation	↑ HIF-1α, CXCR4 and PI3K/AKT	[68]
MCAO/rats	OM-MSCs approximately 1 × 10^6^	Hypoxia 3% O2	↓ apoptosis ↑ motor function	↑ GRP78 and Bcl-2 through miR-181a	[58]
ICH/mice	OM-MSCs 5 × 10^5^	Hypoxia 3% O_2_ and normoxia 21% O_2_	↓ apoptosis and behavioral deficits	-	[59]
ICH/mice	OM-MSCs 2–4 × 10^5^	Hypoxia 3% O_2_ and normoxia 21% O_2_	↓ microglial pyroptosis	-	[69]
SCI/rats	UC-MSCs a total of 10^5^ cells	Physioxy 5% O_2_	↑ axonal regeneration↓ neuroinflammation and apoptosis	↑ BDNF, VEGF and HGF	[70]
EAE/mice	hPDLSCs CM 1.0 mg/mouse	Hypoxia 3% O_2_	↓ inflammation, oxidative stress and apoptosis	↑ IL-37, PI3K/Akt/mTOR signaling	[60]
TBI/rats	AD-MSCs 1 × 10^6^ cells	Hypoxia 2.5% O_2_	↑ cell survival ↓ apoptosis and neuroinflammation	-	[71]
Optic nerve compression/rats	HP-MSCs 2 × 10^6^ cells	Hypoxia 2.2% O_2_	↑ axonal repair	↑ HIF-1α/VEGF signaling	[61]

MCAO: middle cerebral artery occlusion; BM-MSCs: bone marrow-derived MSCs; ↑: increase; ↓: decrease; HIF-1α: hypoxia inducible factor-1α; TBI: traumatic brain injury; CM-conditioned medium; VEGF: vascular endothelial growth factors; HGF: hepatocyte growth factor; BMMSCs-CM: bone marrow derived MSCs-conditioned medium; HI: hypoxia-ischemia; CXCR4: CXC motif chemokine receptor type 4; CXCR12: CXC motif chemokine receptor type 12; SCI: spinal cord injury; MSCs-EVs: MSCs-extracellular vesicles; OM-MSCs: olfactory mucosal derived-MSCs; ICH: intracerebral hemorrhage; bcl-2: B cell lymphoma-2; UC-MSCs: umbilical cord-mesenchymal stromal cells; BDNF: brain-derived neurotrophic factor; EAE: experimental autoimmune encephalomyelitis; hPDLSCs: human periodontal ligament stem cells; IL-37: interleuchin-37; PI3K: phosphatidylinositol 3-kinase; AD-MSCs: adipose-derived MSCs; HP-MSCs: human placenta-derived MSCs.

**Table 4:** Summary of the in vitro studies that described the effects of MSC preconditioning with chemical, pharmacological agents, trophic factors and cytokines.

Cell and Stress	MSCs	Preconditioning	Results	Mechanism	Ref.
NA	hMSCs HS5 cells	BME, T3 and CUR	BME 1 mM: ↑ NPCs differentiation and toxicity T3 0.5 µM: ↑ NPCs differentiation CUR 5 µM: ↑ NPCs differentiation	↑ PAX6, SOX2, DLX2 and GAP-43	[73]
Neuron-like PC12 cells cultured in H/SD co-cultured with BM-MSCs	BM-MSCs	2 h Sevofluorane	↑ cell survival and migration ↓ apoptosis	↑ HIF-1α, HIF-2α, p-Akt/Akt and VEGF	[74]
Cortical neurons exposed to OGD	WJ-MSCs	thrombin	↓ apoptosis, neuroinflammation and oxidative stress	↑ VEGF and BDNF	[75]

hMSCs: human mesenchymal stromal cells; ↑: increase; ↓: decrease; BME: b-mercaptoethanol; T3: triiodothyronine; CUR: curcumin; NPCs: neural precursor cells; BM-MSCs: bone marrow-derived MSCs; HIF-1α: hypoxia inducible factor-1α; HIF-2α: hypoxia inducible factor-2α; VEGF: vascular endothelial growth factors; OGD: oxygen and glucose deprivation; WJ-MSCs: Wharton’s jelly-derived MSCs; BDNF: brain-derived neurotrophic factor.

**Table 5:** Summary of the in vivo studies that described the effects of preconditioning MSCs with chemical, pharmacological agents, trophic factors and cytokines.

Model/Species	MSCs	Preconditioning	Results	Mechanism	Ref.
MCAO/rats	MSCs 3 × 10^6^ per kg	Co-culture with rat cortical neurons	↑ functional recovery ↓ infarction volume	↑ paracrine effects mitochondrial transfer	[76]
AD/rats	MSCs	Dimethyloxalylglycine 0, 250, 500, 750 and 1000 μM	↓ learning and memory deficits	↑ HIF-1	[77]
HI/mice	MSCs-EVs 100 μg EVs dissolved in 50 μL PBS (1.5 × 10^8^ particles)	H_2_S-preconditioned EVs	↓ neuroinflammation, neurodegeneration, cognitive and memory deficits	↑miR-7b-5p that target FOS gene	[78]
3xTg-AD/mice	MSCs-EVs 300 μg/mL	TNFα 20 ng/mL and IFNγ 25 ng/mL	↑ dendritic spines ↓ neuroinflammation	microglia polarization to an anti-inflammatory phenotype	[79]
6-OHDA/rats	BM-MSCs (5 × 10^4^ cells/μL in 4 uL)	Cultured cells with or without FBS supplemented with EGF and bFGF	↑ activity of dopaminergic neurons	↑ BDNF	[80]
N171-82Q HD transgenic mice	BM-MSCs 3.0 × 10^5^ cells	Lithium and VPA	↑ cell survival ↓ apoptosis, huntingtin aggregates, motor and behavioral deficits	↑ expression of trophic, antioxidant, anti-apoptotic factors, cyto-kine/chemokine receptor, migration, cellular stress and mitochondrial energy pathways	[82]
MCAO/reperfusion rats	BM-MSCs 2 × 10^6^ cells per ml dissolved in 1 mL PSB	Sodium hydrosulfide	↑ secretion of growth factor ↓ apoptosis	↑ BDNF and VEGF ↓ Bax/Bcl-2 ratio	[83]
ICH/rats	hPDMSCs 1 × 10^6^ cells/animals	Apocynin	↓ neurodegeneration, cerebral edema	-	[84]
6-OHDA/rats	hPMSCs (2 × 1.5 × 10^5^/rat) or hpNPCs	Proliferation medium containing FGF-4 and heparin	↑ cell differentiation ↓ motor deficits	↑ Delta-like ligand 1 and paracrine factors	[85]
SCI/mice	AT-MSCs 3 μL/site	Growth factors at a dose of 10 ng /mL, retinoic acid and sonic hedgehog for 24 h	↑cell survival, integration and differentiation into neuronal cells ↓ locomotor deficits	-	[86]
HIE/rats	WJ-MSCs 1 × 10^5^	thrombin	↓ apoptosis, neuroinflammation	-	[75]

MCAO: middle cerebral artery occlusion; MSCs: mesenchymal stromal cells; ↑: increase; ↓: decrease; AD: Alzheimer’s disease; HIF-1: hypoxia inducible factor-1; HI: hypoxia-ischemic; MSCs-EVs: MSCs-extracellular vesicles; H_2_S: hydrogen sulfide; TNFα: tumor necrosis factor- α; IFN-γ: interferon-γ; 6-OHDA: 6-hydroxydopamine; bFGF: basic fibroblast growth factor; BM-MSCs: bone marrow-derived MSCs; BDNF: brain-derived neurotrophic factor; HD: Huntington’s disease; VPA: valproic acid; VEGF: vascular endothelial growth factors; bcl-2: B cell lymphoma-2; ICH: intracerebral hemorrhage; hPDMSCs: human placenta-MSCs; FGF-4: fibroblast growth factor-4; hpNPCs: hpMSCs-derived neural precursor cells; SCI: spinal cord injury; AT-MSCs: AT-derived MSCs.

## Data Availability

No new data were created or analyzed in this study. Data sharing is not applicable to this article.

## References

[1] Mushahary D., Spittler A., Kasper C., Weber V., Charwat V. (2018). Isolation, cultivation, and characterization of human mesenchymal stem cells. Cytome. Pt. A J. Int. Soc. Anal. Cytol..

[2] Viswanathan S., Shi Y., Galipeau J., Krampera M., Leblanc K., Martin I., Nolta J., Phinney D.G., Sensebe L. (2019). Mesenchymal stem versus stromal cells: International Society for Cell & Gene Therapy (ISCT(R)) Mesenchymal Stromal Cell committee position statement on nomenclature. Cytotherapy.

[3] Dominici M., Le Blanc K., Mueller I., Slaper-Cortenbach I., Marini F., Krause D., Deans R., Keating A., Prockop D., Horwitz E. (2006). Minimal criteria for defining multipotent mesenchymal stromal cells. The International Society for Cellular Therapy position statement. Cytotherapy.

[4] Strioga M., Viswanathan S., Darinskas A., Slaby O., Michalek J. (2012). Same or not the same? Comparison of adipose tissue-derived versus bone marrow-derived mesenchymal stem and stromal cells. Stem Cells Dev..

[5] Bajek A., Gurtowska N., Olkowska J., Kazmierski L., Maj M., Drewa T. (2016). Adipose-Derived Stem Cells as a Tool in Cell-Based Therapies. Arch. Immunol. Ther. Exp..

[6] Berebichez-Fridman R., Montero-Olvera P.R. (2018). Sources and Clinical Applications of Mesenchymal Stem Cells: State-of-the-art review. Sult. Qaboos Univ. Med. J..

[7] Kwon A., Kim Y., Kim M., Kim J., Choi H., Jekarl D.W., Lee S., Kim J.M., Shin J.C., Park I.Y. (2016). Tissue-specific Differentiation Potency of Mesenchymal Stromal Cells from Perinatal Tissues. Sci. Rep..

[8] Xiao L., Tsutsui T. (2013). Human dental mesenchymal stem cells and neural regeneration. Hum. Cell.

[9] Park Y.J., Cha S., Park Y.S. (2016). Regenerative Applications Using Tooth Derived Stem Cells in Other Than Tooth Regeneration: A Literature Review. Stem Cells Int..

[10] Pittenger M.F., Mackay A.M., Beck S.C., Jaiswal R.K., Douglas R., Mosca J.D., Moorman M.A., Simonetti D.W., Craig S., Marshak D.R. (1999). Multilineage potential of adult human mesenchymal stem cells. Science.

[11] Harrell C.R., Fellabaum C., Jovicic N., Djonov V., Arsenijevic N., Volarevic V. (2019). Molecular Mechanisms Responsible for Therapeutic Potential of Mesenchymal Stem Cell-Derived Secretome. Cells.

[12] Uccelli A., Moretta L., Pistoia V. (2008). Mesenchymal stem cells in health and disease. Nat. Rev. Immunol..

[13] Mahmood A., Lu D., Chopp M. (2004). Intravenous administration of marrow stromal cells (MSCs) increases the expression of growth factors in rat brain after traumatic brain injury. J. Neurotrauma.

[14] Wei X., Yang X., Han Z.P., Qu F.F., Shao L., Shi Y.F. (2013). Mesenchymal stem cells: A new trend for cell therapy. Acta Pharmacol. Sin..

[15] Vizoso F.J., Eiro N., Cid S., Schneider J., Perez-Fernandez R. (2017). Mesenchymal Stem Cell Secretome: Toward Cell-Free Therapeutic Strategies in Regenerative Medicine. Int. J. Mol. Sci..

[16] Eleuteri S., Fierabracci A. (2019). Insights into the Secretome of Mesenchymal Stem Cells and Its Potential Applications. Int. J. Mol. Sci..

[17] Hasan A., Deeb G., Rahal R., Atwi K., Mondello S., Marei H.E., Gali A., Sleiman E. (2017). Mesenchymal Stem Cells in the Treatment of Traumatic Brain Injury. Front. Neurol..

[18] Wang Z., Luo Y., Chen L., Liang W. (2017). Safety of neural stem cell transplantation in patients with severe traumatic brain injury. Exp. Ther. Med..

[19] Paul G., Anisimov S.V. (2013). The secretome of mesenchymal stem cells: Potential implications for neuroregeneration. Biochimie.

[20] Hu C., Li L. (2018). Preconditioning influences mesenchymal stem cell properties in vitro and in vivo. J. Cell. Mol. Med..

[21] Teli P., Kale V., Vaidya A. (2021). Extracellular vesicles isolated from mesenchymal stromal cells primed with neurotrophic factors and signaling modifiers as potential therapeutics for neurodegenerative diseases. Curr. Res. Trans. Med..

[22] Sheikh S., Safia, Haque E., Mir S.S. (2013). Neurodegenerative Diseases: Multifactorial Conformational Diseases and Their Therapeutic Interventions. J. Neurodegener. Dis..

[23] Sakthiswary R., Raymond A.A. (2012). Stem cell therapy in neurodegenerative diseases: From principles to practice. Neural Regen. Res..

[24] Dihne M., Bernreuther C., Hagel C., Wesche K.O., Schachner M. (2006). Embryonic stem cell-derived neuronally committed precursor cells with reduced teratoma formation after transplantation into the lesioned adult mouse brain. Stem Cells.

[25] Tao H., Chen X., Wei A., Song X., Wang W., Liang L., Zhao Q., Han Z., Han Z., Wang X. (2018). Comparison of Teratoma Formation between Embryonic Stem Cells and Parthenogenetic Embryonic Stem Cells by Molecular Imaging. Stem Cells Int..

[26] Urrutia D.N., Caviedes P., Mardones R., Minguell J.J., Vega-Letter A.M., Jofre C.M. (2019). Comparative study of the neural differentiation capacity of mesenchymal stromal cells from different tissue sources: An approach for their use in neural regeneration therapies. PLoS ONE.

[27] Prpar Mihevc S., Kokondoska Grgich V., Kopitar A.N., Mohoric L., Majdic G. (2020). Neural differentiation of canine mesenchymal stem cells/multipotent mesenchymal stromal cells. BMC Vet. Res..

[28] Heng B.C., Lim L.W., Wu W., Zhang C. (2016). An Overview of Protocols for the Neural Induction of Dental and Oral Stem Cells In Vitro. Tissue Eng. Pt. B Rev..

[29] Franco Lambert A.P., Fraga Zandonai A., Bonatto D., Cantarelli Machado D., Pegas Henriques J.A. (2009). Differentiation of human adipose-derived adult stem cells into neuronal tissue: Does it work?. Differentiat. Res. Biol. Divers..

[30] Ferroni L., Gardin C., Tocco I., Epis R., Casadei A., Vindigni V., Mucci G., Zavan B. (2013). Potential for neural differentiation of mesenchymal stem cells. Adv. Biochem. Eng. Biotechnol..

[31] Bernardo M.E., Pagliara D., Locatelli F. (2012). Mesenchymal stromal cell therapy: A revolution in Regenerative Medicine?. Bone Marrow Transplant..

[32] Li X., Guan Y., Li C., Zhang T., Meng F., Zhang J., Li J., Chen S., Wang Q., Wang Y. (2022). Immunomodulatory effects of mesenchymal stem cells in peripheral nerve injury. Stem Cell Res. Ther..

[33] Marconi S., Bonaconsa M., Scambi I., Squintani G.M., Rui W., Turano E., Ungaro D., D’Agostino S., Barbieri F., Angiari S. (2013). Systemic treatment with adipose-derived mesenchymal stem cells ameliorates clinical and pathological features in the amyotrophic lateral sclerosis murine model. Neuroscience.

[34] Zhu J., Liu Q., Jiang Y., Wu L., Xu G., Liu X. (2015). Enhanced angiogenesis promoted by human umbilical mesenchymal stem cell transplantation in stroked mouse is Notch1 signaling associated. Neuroscience.

[35] Zhou L., Lin Q., Wang P., Yao L., Leong K., Tan Z., Huang Z. (2017). Enhanced neuroprotective efficacy of bone marrow mesenchymal stem cells co-overexpressing BDNF and VEGF in a rat model of cardiac arrest-induced global cerebral ischemia. Cell Death Dis..

[36] Sart S., Ma T., Li Y. (2014). Preconditioning stem cells for in vivo delivery. BioRes. Open Access.

[37] Ferreira J.R., Teixeira G.Q., Santos S.G., Barbosa M.A., Almeida-Porada G., Goncalves R.M. (2018). Mesenchymal Stromal Cell Secretome: Influencing Therapeutic Potential by Cellular Pre-conditioning. Front. Immunol..

[38] Noronha N.C., Mizukami A., Caliari-Oliveira C., Cominal J.G., Rocha J.L.M., Covas D.T., Swiech K., Malmegrim K.C.R. (2019). Priming approaches to improve the efficacy of mesenchymal stromal cell-based therapies. Stem Cell Res. Ther..

[39] Saparov A., Ogay V., Nurgozhin T., Jumabay M., Chen W.C. (2016). Preconditioning of Human Mesenchymal Stem Cells to Enhance Their Regulation of the Immune Response. Stem Cells Int..

[40] Xia Y., Choi H.K., Lee K. (2012). Recent advances in hypoxia-inducible factor (HIF)-1 inhibitors. Eur. J. Med. Chem..

[41] Ezashi T., Das P., Roberts R.M. (2005). Low O_2_ tensions and the prevention of differentiation of hES cells. Proc. Natl. Acad. Sci. USA.

[42] Simon M.C., Keith B. (2008). The role of oxygen availability in embryonic development and stem cell function. Nat. Rev. Mol. Cell Biol..

[43] Vieira H.L., Alves P.M., Vercelli A. (2011). Modulation of neuronal stem cell differentiation by hypoxia and reactive oxygen species. Prog. Neurobiol..

[44] Mung K.L., Tsui Y.P., Tai E.W., Chan Y.S., Shum D.K., Shea G.K. (2016). Rapid and efficient generation of neural progenitors from adult bone marrow stromal cells by hypoxic preconditioning. Stem Cell Res. Ther..

[45] Theus M.H., Wei L., Cui L., Francis K., Hu X., Keogh C., Yu S.P. (2008). In vitro hypoxic preconditioning of embryonic stem cells as a strategy of promoting cell survival and functional benefits after transplantation into the ischemic rat brain. Exp. Neurol..

[46] Zhang Z., Alexanian A.R. (2012). Dopaminergic-like cells from epigenetically reprogrammed mesenchymal stem cells. J. Cell. Mol. Med..

[47] Pacary E., Legros H., Valable S., Duchatelle P., Lecocq M., Petit E., Nicole O., Bernaudin M. (2006). Synergistic effects of CoCl(2) and ROCK inhibition on mesenchymal stem cell differentiation into neuron-like cells. J. Cell Sci..

[48] Pacary E., Tixier E., Coulet F., Roussel S., Petit E., Bernaudin M. (2007). Crosstalk between HIF-1 and ROCK pathways in neuronal differentiation of mesenchymal stem cells, neurospheres and in PC12 neurite outgrowth. Mol. Cell. Neurosci..

[49] Gugliandolo A., Diomede F., Scionti D., Bramanti P., Trubiani O., Mazzon E. (2019). The Role of Hypoxia on the Neuronal Differentiation of Gingival Mesenchymal Stem Cells: A Transcriptional Study. Cell Transplant..

[50] Teixeira F.G., Panchalingam K.M., Anjo S.I., Manadas B., Pereira R., Sousa N., Salgado A.J., Behie L.A. (2015). Do hypoxia/normoxia culturing conditions change the neuroregulatory profile of Wharton Jelly mesenchymal stem cell secretome?. Stem Cell Res. Ther..

[51] Kim Y.S., Noh M.Y., Cho K.A., Kim H., Kwon M.S., Kim K.S., Kim J., Koh S.H., Kim S.H. (2015). Hypoxia/Reoxygenation-Preconditioned Human Bone Marrow-Derived Mesenchymal Stromal Cells Rescue Ischemic Rat Cortical Neurons by Enhancing Trophic Factor Release. Mol. Neurobiol..

[52] Chen J., Yang Y., Shen L., Ding W., Chen X., Wu E., Cai K., Wang G. (2017). Hypoxic Preconditioning Augments the Therapeutic Efficacy of Bone Marrow Stromal Cells in a Rat Ischemic Stroke Model. Cell. Mol. Neurobiol..

[53] Yu H., Xu Z., Qu G., Wang H., Lin L., Li X., Xie X., Lei Y., He X., Chen Y. (2021). Hypoxic Preconditioning Enhances the Efficacy of Mesenchymal Stem Cells-Derived Conditioned Medium in Switching Microglia toward Anti-inflammatory Polarization in Ischemia/Reperfusion. Cell. Mol. Neurobiol..

[54] Luo Z., Wu F., Xue E., Huang L., Yan P., Pan X., Zhou Y. (2019). Hypoxia preconditioning promotes bone marrow mesenchymal stem cells survival by inducing HIF-1alpha in injured neuronal cells derived exosomes culture system. Cell Death Dis..

[55] Gregorius J., Wang C., Stambouli O., Hussner T., Qi Y., Tertel T., Borger V., Mohamud Yusuf A., Hagemann N., Yin D. (2021). Small extracellular vesicles obtained from hypoxic mesenchymal stromal cells have unique characteristics that promote cerebral angiogenesis, brain remodeling and neurological recovery after focal cerebral ischemia in mice. Basic Res. Cardiol..

[56] Lech W., Sarnowska A., Kuczynska Z., Dabrowski F., Figiel-Dabrowska A., Domanska-Janik K., Buzanska L., Zychowicz M. (2020). Biomimetic microenvironmental preconditioning enhance neuroprotective properties of human mesenchymal stem cells derived from Wharton’s Jelly (WJ-MSCs). Sci. Rep..

[57] Huang Y., Liu Z., Tan F., Hu Z., Lu M. (2020). Effects of the Insulted Neuronal Cells-Derived Extracellular Vesicles on the Survival of Umbilical Cord-Derived Mesenchymal Stem Cells following Cerebral Ischemia/Reperfusion Injury. Oxid. Med. Cell. Longev..

[58] Zhuo Y., Chen W., Li W., Huang Y., Duan D., Ge L., He J., Liu J., Hu Z., Lu M. (2021). Ischemic-hypoxic preconditioning enhances the mitochondrial function recovery of transplanted olfactory mucosa mesenchymal stem cells via miR-181a signaling in ischemic stroke. Aging.

[59] Liu J., He J., Ge L., Xiao H., Huang Y., Zeng L., Jiang Z., Lu M., Hu Z. (2021). Hypoxic preconditioning rejuvenates mesenchymal stem cells and enhances neuroprotection following intracerebral hemorrhage via the miR-326-mediated autophagy. Stem Cell Res. Ther..

[60] Giacoppo S., Thangavelu S.R., Diomede F., Bramanti P., Conti P., Trubiani O., Mazzon E. (2017). Anti-inflammatory effects of hypoxia-preconditioned human periodontal ligament cell secretome in an experimental model of multiple sclerosis: A key role of IL-37. FASEB J..

[61] Kwon H., Park M., Nepali S., Lew H. (2020). Hypoxia-Preconditioned Placenta-Derived Mesenchymal Stem Cells Rescue Optic Nerve Axons Via Differential Roles of Vascular Endothelial Growth Factor in an Optic Nerve Compression Animal Model. Mol. Neurobiol..

[62] Wei L., Fraser J.L., Lu Z.Y., Hu X., Yu S.P. (2012). Transplantation of hypoxia preconditioned bone marrow mesenchymal stem cells enhances angiogenesis and neurogenesis after cerebral ischemia in rats. Neurobiol. Dis..

[63] Chang C.P., Chio C.C., Cheong C.U., Chao C.M., Cheng B.C., Lin M.T. (2013). Hypoxic preconditioning enhances the therapeutic potential of the secretome from cultured human mesenchymal stem cells in experimental traumatic brain injury. Clin. Sci..

[64] Wei Z.Z., Gu X., Ferdinand A., Lee J.H., Ji X., Ji X.M., Yu S.P., Wei L. (2015). Intranasal delivery of bone marrow mesenchymal stem cells improved neurovascular regeneration and rescued neuropsychiatric deficits after neonatal stroke in rats. Cell Transplant..

[65] Roth S., Dreixler J.C., Mathew B., Balyasnikova I., Mann J.R., Boddapati V., Xue L., Lesniak M.S. (2016). Hypoxic-Preconditioned Bone Marrow Stem Cell Medium Significantly Improves Outcome After Retinal Ischemia in Rats. Investig. Ophthalmol. Vis. Sci..

[66] Dai Y., Li W., Zhong M., Chen J., Cheng Q., Liu Y., Li T. (2017). The paracrine effect of cobalt chloride on BMSCs during cognitive function rescue in the HIBD rat. Behav. Brain Res..

[67] Hu Y., Chen W., Wu L., Jiang L., Qin H., Tang N. (2019). Hypoxic preconditioning improves the survival and neural effects of transplanted mesenchymal stem cells via CXCL12/CXCR4 signalling in a rat model of cerebral infarction. Cell Biochem. Funct..

[68] Wang J.W., Qiu Y.R., Fu Y., Liu J., He Z.J., Huang Z.T. (2017). Transplantation with hypoxia-preconditioned mesenchymal stem cells suppresses brain injury caused by cardiac arrest-induced global cerebral ischemia in rats. J. Neurosci. Res..

[69] Liu J., He J., Huang Y., Ge L., Xiao H., Zeng L., Jiang Z., Lu M., Hu Z. (2021). Hypoxia-preconditioned mesenchymal stem cells attenuate microglial pyroptosis after intracerebral hemorrhage. Ann. Trans. Med..

[70] Zhilai Z., Biling M., Sujun Q., Chao D., Benchao S., Shuai H., Shun Y., Hui Z. (2016). Preconditioning in lowered oxygen enhances the therapeutic potential of human umbilical mesenchymal stem cells in a rat model of spinal cord injury. Brain Res..

[71] Ma H., Lam P.K., Tong C.S.W., Lo K.K.Y., Wong G.K.C., Poon W.S. (2019). The neuroprotection of hypoxic adipose tissue-derived mesenchymal stem cells in experimental traumatic brain injury. Cell Transplant..

[72] Talwadekar M., Fernandes S., Kale V., Limaye L. (2017). Valproic acid enhances the neural differentiation of human placenta derived-mesenchymal stem cells in vitro. J. Tissue Eng. Regen. Med..

[73] Sallam A., Sudha T., Darwish N.H.E., Eghotny S.A.E.D., Hassaan P.S., Mousa S.A. (2021). In vitro differentiation of human bone marrow stromal cells into neural precursor cells using small molecules. J. Neurosci. Methods.

[74] Sun X., Fang B., Zhao X., Zhang G., Ma H. (2014). Preconditioning of mesenchymal stem cells by sevoflurane to improve their therapeutic potential. PLoS ONE.

[75] Kim Y.E., Sung S.I., Chang Y.S., Ahn S.Y., Sung D.K., Park W.S. (2019). Thrombin Preconditioning Enhances Therapeutic Efficacy of Human Wharton’s Jelly-Derived Mesenchymal Stem Cells in Severe Neonatal Hypoxic Ischemic Encephalopathy. Int. J. Mol. Sci..

[76] Babenko V.A., Silachev D.N., Zorova L.D., Pevzner I.B., Khutornenko A.A., Plotnikov E.Y., Sukhikh G.T., Zorov D.B. (2015). Improving the Post-Stroke Therapeutic Potency of Mesenchymal Multipotent Stromal Cells by Cocultivation With Cortical Neurons: The Role of Crosstalk Between Cells. Stem Cells Transl. Med..

[77] Esmaeilzade B., Artimani T., Amiri I., Najafi R., Shahidi S., Sabec M., Farzadinia P., Zare M., Zahiri M., Soleimani Asl S. (2019). Dimethyloxalylglycine preconditioning enhances protective effects of bone marrow-derived mesenchymal stem cells in Abeta- induced Alzheimer disease. Physiol. Behav..

[78] Chu X., Liu D., Li T., Ke H., Xin D., Wang S., Cao Y., Xue H., Wang Z. (2020). Hydrogen sulfide-modified extracellular vesicles from mesenchymal stem cells for treatment of hypoxic-ischemic brain injury. J. Control. Release.

[79] Losurdo M., Pedrazzoli M., D’Agostino C., Elia C.A., Massenzio F., Lonati E., Mauri M., Rizzi L., Molteni L., Bresciani E. (2020). Intranasal delivery of mesenchymal stem cell-derived extracellular vesicles exerts immunomodulatory and neuroprotective effects in a 3xTg model of Alzheimer’s disease. Stem Cells Transl. Med..

[80] Somoza R., Juri C., Baes M., Wyneken U., Rubio F.J. (2010). Intranigral transplantation of epigenetically induced BDNF-secreting human mesenchymal stem cells: Implications for cell-based therapies in Parkinson’s disease. Biol. Blood Marrow Transplant..

[81] Chiu C.T., Wang Z., Hunsberger J.G., Chuang D.M. (2013). Therapeutic potential of mood stabilizers lithium and valproic acid: Beyond bipolar disorder. Pharmacol. Rev..

[82] Linares G.R., Chiu C.T., Scheuing L., Leng Y., Liao H.M., Maric D., Chuang D.M. (2016). Preconditioning mesenchymal stem cells with the mood stabilizers lithium and valproic acid enhances therapeutic efficacy in a mouse model of Huntington’s disease. Exp. Neurol..

[83] Zhang Q., Liu S., Li T., Yuan L., Liu H., Wang X., Wang F., Wang S., Hao A., Liu D. (2016). Preconditioning of bone marrow mesenchymal stem cells with hydrogen sulfide improves their therapeutic potential. Oncotarget.

[84] Min S., Kim O.J., Bae J., Chung T.N. (2018). Effect of Pretreatment with the NADPH Oxidase Inhibitor Apocynin on the Therapeutic Efficacy of Human Placenta-Derived Mesenchymal Stem Cells in Intracerebral Hemorrhage. Int. J. Mol. Sci..

[85] Kim H.W., Lee H.S., Kang J.M., Bae S.H., Kim C., Lee S.H., Schwarz J., Kim G.J., Kim J.S., Cha D.H. (2018). Dual Effects of Human Placenta-Derived Neural Cells on Neuroprotection and the Inhibition of Neuroinflammation in a Rodent Model of Parkinson’s Disease. Cell Transplant..

[86] Gao S., Guo X., Zhao S., Jin Y., Zhou F., Yuan P., Cao L., Wang J., Qiu Y., Sun C. (2019). Differentiation of human adipose-derived stem cells into neuron/motoneuron-like cells for cell replacement therapy of spinal cord injury. Cell Death Dis..

[87] Caplan H., Olson S.D., Kumar A., George M., Prabhakara K.S., Wenzel P., Bedi S., Toledano-Furman N.E., Triolo F., Kamhieh-Milz J. (2019). Mesenchymal Stromal Cell Therapeutic Delivery: Translational Challenges to Clinical Application. Front. Immunol..

[88] Bongso A., Fong C.Y., Gauthaman K. (2008). Taking stem cells to the clinic: Major challenges. J. Cell. Biochem..

[89] Chrostek M.R., Fellows E.G., Crane A.T., Grande A.W., Low W.C. (2019). Efficacy of stem cell-based therapies for stroke. Brain Res..

[90] Liu S., Zhou J., Zhang X., Liu Y., Chen J., Hu B., Song J., Zhang Y. (2016). Strategies to Optimize Adult Stem Cell Therapy for Tissue Regeneration. Int. J. Mol. Sci..

[91] Qazi T.H., Mooney D.J., Duda G.N., Geissler S. (2017). Biomaterials that promote cell-cell interactions enhance the paracrine function of MSCs. Biomaterials.

[92] Shrestha B., Coykendall K., Li Y., Moon A., Priyadarshani P., Yao L. (2014). Repair of injured spinal cord using biomaterial scaffolds and stem cells. Stem Cell Res. Ther..

[93] Braid L.R., Wood C.A., Wiese D.M., Ford B.N. (2018). Intramuscular administration potentiates extended dwell time of mesenchymal stromal cells compared to other routes. Cytotherapy.

[94] Nijboer C.H., Kooijman E., van Velthoven C.T., van Tilborg E., Tiebosch I.A., Eijkelkamp N., Dijkhuizen R.M., Kesecioglu J., Heijnen C.J. (2018). Intranasal Stem Cell Treatment as a Novel Therapy for Subarachnoid Hemorrhage. Stem Cells Dev..

[95] Danielyan L., Schafer R., von Ameln-Mayerhofer A., Bernhard F., Verleysdonk S., Buadze M., Lourhmati A., Klopfer T., Schaumann F., Schmid B. (2011). Therapeutic efficacy of intranasally delivered mesenchymal stem cells in a rat model of Parkinson disease. Rejuvenat. Res..

[96] Salama M., Sobh M., Emam M., Abdalla A., Sabry D., El-Gamal M., Lotfy A., El-Husseiny M., Sobh M., Shalash A. (2017). Effect of intranasal stem cell administration on the nigrostriatal system in a mouse model of Parkinson’s disease. Exp. Ther. Med..

[97] Elliott Donaghue I., Tam R., Sefton M.V., Shoichet M.S. (2014). Cell and biomolecule delivery for tissue repair and regeneration in the central nervous system. J. Control. Release.

[98] Lalu M.M., Mazzarello S., Zlepnig J., Dong Y.Y.R., Montroy J., McIntyre L., Devereaux P.J., Stewart D.J., David Mazer C., Barron C.C. (2018). Safety and Efficacy of Adult Stem Cell Therapy for Acute Myocardial Infarction and Ischemic Heart Failure (SafeCell Heart): A Systematic Review and Meta-Analysis. Stem Cells Transl. Med..

[99] von Bahr L., Batsis I., Moll G., Hagg M., Szakos A., Sundberg B., Uzunel M., Ringden O., Le Blanc K. (2012). Analysis of tissues following mesenchymal stromal cell therapy in humans indicates limited long-term engraftment and no ectopic tissue formation. Stem Cells.

[100] Cruz F.F., Rocco P.R.M. (2020). The potential of mesenchymal stem cell therapy for chronic lung disease. Expert Rev. Respir. Med..

